# Progress in Surgery

**Published:** 1899-09-30

**Authors:** 


### PROGRESS IN SURGERY.

### OTOLOGY.

(*Continued from page 443.*)

**Cerebral and Cerebellar Abscesses.**—H. Tilley[4] reports a case of abscess of the tempero-sphenoidal lobe successfully treated. A girl, nine years of age, had had otorrhœa for four years. The discharge having suddenly stopped, the patient was admitted to hospital. She had earache, headache, looked very ill, had wide pupils, and slow cerebration, but no optic neuritis. A week later there was a free flow of pus from the ear, during an examination. From the quantity it seemed clear that there was communication with an intracranial abscess. The mastoid was cleared of granulation tissue and pus, the roof of the antrum was eroded, and through it a Lister's sinus forceps was passed an inch and a-half into the brain, and when dilated a free flow of pus ensued. A rubber tube was passed in, and the patient made ultimately a good recovery. At the meeting of the American Otological Society in June, 1898,[3] Dr. J. Orne Green showed three specimens of caries of the temporal bone. In all the fatal result was due to abscess in the antero inferior portion of the cerebellum, the brain being reached in two cases through the meatus internus, and in one through the aquæductus vestibuli. In all three cases the tympanic wall of the vestibule was perforated by caries, and the external semi-circular canal opened into. At the seventieth Congress of German Physicians in September, 1898, Professor Hausburg[3] reported two cases of cerebellar abscess. The first was in a boy of 12, who had had otorrhœa for 18 months. There was headache, vertigo, vomiting, rigidity of the neck, and tenderness of the mastoid. The mastoid was found at the first operation carious, and there was a large extradural abscess. Four days later the posterior part of the mastoid was removed, and a large medial abscess in the cerebellum evacuated. Recovery ensued. The second case was a man of 22. There had been otorrhœa since childhood. He was seized with vomiting, dizziness, and occipital headache, but no fever. Nine days later there was somnolence, prostration, difference in the pupils, left-sided ptosis, rigidity of the neck, Cheyne-Stokes breathing, and a small irregular pulse. Operation was attempted, but the patient died in twenty minutes. An autopsy showed a large abscess which had almost entirely destroyed the right cerebellar hemisphere. There was free communication between the sinus and the abscess cavity, and the ends of the sinus were thromboid. No fistula led to the mastoid. An instructive case is recorded by R. Lake in the St. Thomas's Hospital Reports for 1897.[3] A man of 31 for 19 years had had otorrhœa and deafness on the left side. A typical Stacke's operation was done. Facial paralysis was noted after. He went on well until the tenth day, when the temperature went up and headache came on. On the thirteenth day there was inability to read, loss of memory for names of people and places, and marked giddiness. Later there was a brisker knee jerk on the right side, and greater weakness of the grasp on the right. Mr. Ballance then evacuated fœtid pus from the left temporo-sphenoidal lobe. The abscess was acute, there being no thick wall. Rubber drain tubes were used. A month later the cerebral symptoms had almost completely disappeared. At the end of six months also the facial paralysis had disappeared. It had been treated by the use of the interrupted current.

**Meningitis.**—Milligan[5] has referred to the value of lumbar puncture in the diagnosis of doubtful cases of ear disease in which meningitis is suspected, either as the only complication, or with others. Normal cerebro-spinal fluid contains the merest trace of albumin Where cerebral abscess or tumour exists the albumin is slightly increased, but in meningitis it is markedly increased. Granert speaks highly of this method of diagnosing meningitis. In addition to albumin, the fluid often contains bacteria (meningo-cocci, pneumo-cocci, strepto-cocci, &c.). Positive results are alone of value. Geronzi[5] reports a case of meningitis which followed the removal of aural polypus. It was in a boy of 12, who had otorrhœa from infancy. For a month there had been headache. A large polyp was removed with a snare, and the boy sent home. The same night headache and fever increased, and next morning there was delirium. Death resulted in eight days. Post-mortem there was a circumscribed meningitis and encephalitis over the mastoid antrum. The case serves to illustrate the possibility of danger arising from small operations about the ear. Waldvogel reports[4] two cases in which he believes that certain cerebral symptoms—somnolence, aphasia, cerebral vomiting, and convulsions—were due to some change in the brain and its membranes which was easily recoverable from, such as hyperæmia with slight exudation. The above symptoms did not disappear for some days after the escape of pus from the ear and fall of temperature. Retention of pus in the tympanum cannot alone cause cerebral symptoms. In serous meningitis the symptoms last longer than in these cases, *i.e.*, more than eight days. It is impossible to tell post-mortem whether slight hyperæmia and œdema existed during life.

**Syphilitic Deafness.**—Pritchard and Cheatle,[2] writing on this subject, point out that the typical mode of onset is a rapidly developed nerve deafness without giddiness, generally preceded, but occasionally followed, by interstitial keratitis. In such cases the probability is that there is periostitic or ostitic thickening of the bony structures of the labyrinth. In rare cases the disease is ushered in by attacks of giddiness of the Ménière type, and may run an acute or chronic course. The cases without giddiness are due to changes analogous to those of tertiary syphilis, whereas the others are very comparable to the lesions of the secondary period.

**Ophthalmoscopy in Diagnosis.**—Gradenigo[6] asserts, from personal experience, that the ocular lesion is sometimes the only pathognomonic sign of the commencement of some intracranial extension of aural disease. He says it is even met with frequently in extradural abscess, which is the primary seat of invasion of inflammation from the ear into the cranial cavity. These ocular lesions vary from congestion of the papilla to optic neuritis. As a rule they are bilateral, but generally more marked on the affected side. They rapidly pass off when the complications are relieved. The author maintains that papillitis is certain evidence of intracranial invasion. On the other hand it is not a constant sign in any of the complications. Of the 172 cases Gradenigo has analysed, papillitis was present in 52·3 per cent. It was most common in cerebellar abscess, simple or complicated with sinus thrombosis; least common (41 per cent.) in uncomplicated extradural abscess. As extradural abscesses give, as a rule, no very clear evidence of their existence, it is important to note that in a large minority papillitis is present. It is remarkable that papillitis may be absent in grave cases of these intracranial complications and present in slight cases.

**Tubercular Ear Disease.**—Kretschmann[6] distinguishes three types of this affection. In the first, in an early stage, there are miliary tubercles on the tympanic membrane, then numerous perforations occur, and later total destruction of the membrane. The tympanic mucous membrane is red, thickened, and ulcerated. There is no pain. This form occurs in cases of advanced tuberculosis. In the second type there is otorrhœa and deafness, but no pain. Granulations quickly fill up the tympanum and the accessory cavities, and the ossicles are exfoliated. Erosion of the internal carotid or jugular vein and facial paralysis may result. The third form is characterised by a necrotic circumscribed spot on the labyrinth wall. Diagnosis is made by finding Koch's bacillus, the clinical appearance, or by microscopic examination. Balsam of Peru and iodoform are used in the early stages; later, operations must be performed.

**Treatment in Otitis Media.**—Goldstein[2] writes on this. When the pus is copious, thick, and ropy, gentle syringing with warm antiseptic fluid is recommended, but in most cases of purulent otitis dry cleansing is found most useful by the author. Where the perforation is large irrigation may force infection into previously healthy parts, and is frequently responsible for mastoid infection. Dry treatment prevents infiltration and softening of the mucous membrane, a condition frequent irrigation favours. It reduces to a minimum the tendency to form polypi. After cleansing nasophen is applied rather than boracic acid or iodoform. The only fluid medicaments the author has used freely are a saturated solution of boracic acid in absolute alcohol, and hydrozone. These are applied with a medicine dropper rather than with a syringe.

[4] Ditto, Jan., 1899. [5] Treatment, Dec., 1898. [6] Jour. of Laryngol., Rhin., and Otology, April, 1899.

